# The Influence of Microstructure on the Flexural Properties of 3D Printed Zirconia Part via Digital Light Processing Technology

**DOI:** 10.3390/ma15041602

**Published:** 2022-02-21

**Authors:** Boran Wang, Ali Arab, Jing Xie, Pengwan Chen

**Affiliations:** 1 State Key Laboratory of Explosion Science and Technology, Beijing Institute of Technology, Beijing 100081, China; brwang@bit.edu.cn (B.W.); pwchen@bit.edu.cn (P.C.); 2 Advanced Technology Research Institute, Beijing Institute of Technology, Jinan 250307, China; 3 Explosion Protection and Emergency Disposal Technology Engineering Research Center of the Ministry of Education, Beijing 100081, China

**Keywords:** zirconia, digital light processing, microstructure, flexural strength, digital image correlation, fractography

## Abstract

In recent years, additive manufacturing of ceramics is becoming of increasing interest due to the possibility of the fabrication of complex shaped parts. However, the fabrication of a fully dense bulk ceramic part without cracks and defects is still challenging. In the presented work, the digital light processing method was introduced for fabricating zirconia parts. The flexural properties of the printed zirconia were systematically investigated via a three-point bending test with the digital image correlation method, scanning electron microscopy observation and fractography analysis. Due to the anisotropy of the sample, the bending deformation behaviors of the zirconia samples in the parallel and vertical printing directions were significantly different. The flexural strength and the related elastic modulus of the samples under vertical loading were higher than that of the parallel loading, as the in-plane strength is higher than that of the interlayer strength. The maximum horizontal strain always appeared at the bottom center before the failure for the parallel loading case; while the maximum horizontal strain for the vertical loading moved upward from the bottom center to the top center. There was a clear dividing line between the minimum perpendicular strain and the maximum perpendicular strain of the samples under parallel loading; however, under vertical loading, the perpendicular strain declined from the bottom to the top along the crack path. The surrounding dense part of the sintered sample (a few hundred microns) was mainly composed of large and straight cracks between printing layers, whereas the interior contained numerous small winding cracks. The intense cracks inside the sample led to a low flexural property compared to other well-prepared zirconia samples, which the inadequate additive formulations would be the main reason for the generation of cracks. A better understanding of the additive formulation (particularly the dispersant) and the debinding-sintering process are necessary for future improvement.

## 1. Introduction

Zirconia (ZrO_2_) is widely used as an implant due to its excellent biocompatibility, mechanical properties, and satisfying aesthetics [1,2]. It has three phases: cubic ZrO_2_ (c-ZrO_2_) exists at temperatures higher than 2370 °C; tetragonal ZrO_2_ (t-ZrO_2_) exists between 1170 and 2370 °C, and when the temperatures are lower than 1170 °C, a phase transition from t- ZrO_2_ to monoclinic ZrO_2_ (m-ZrO_2_) occurs. The t-ZrO_2_ phase, however, can be retained at RT by adding metal oxides, such as yttria (Y_2_O_3_). The mechanical properties of ZrO_2_ are strongly dependent on its phase and grain size. With different fabrication processes and raw materials, a wide range of the mechanical properties could be achieved (e.g., the compression strength of around 2000 MPa, the flexural strength of 800–1200 MPa, and fracture toughness of 5–10 MPam^1/2^).

With traditional manufacturing technology (gel casting, slip casting, dry pressing, injection molding, tape casting, and so on) it is challenging to make ceramic materials into sophisticated shapes. As a result, one cannot take full advantage of their outstanding characteristics. Recently, it has been of great interest to use the additive manufacturing (AM) [3,4] technique in the ceramic industry to sidestep the conventional manufacturing challenges and save potentially up to 80% of the fabrication cost of the ceramic parts [5].

Among the various AM techniques that be used for ceramic parts fabrication (three-dimensional printing (3DP) [6], stereolithography (SLA) [7,8,9], 3D gel-printing [10], a robocasting technique [11], direct inkjet printing (DIP) [12,13] and fused deposition modeling (FDM) [14,15], etc.), digital light processing (DLP) [2,5,14,16,17,18,19,20,21,22] is one of the most promising techniques. The parts fabricated by this technique exhibit fine surface finishes and very high spatial resolution. In this technique, a ceramic slurry is solidified layer by layer via UV-light exposure, and then the printed part is sintered to produce a fully dense sample.

Many researchers have used the DLP method to fabricate ZrO_2_ parts. Osman et al. [2] printed a custom-designed ZrO_2_ implant by DLP with desirable efficiency and dimensional accuracy. In addition, the effects of exposure time, printing angle, properties of slurry and debinding process on the microstructure and physical properties of DLP products were comprehensively investigated by different researchers [14,16,17]. A novel DLP-stereolithography-based 3D printing process was developed to fabricate complex ZrO_2_ parts either dense or with internal architecture [18,19,20]. It was found that the ZrO_2_ stabilized with 3 mol.% yttria was more favorable for slurry preparation compared to the ZrO_2_ stabilized with 8 mol.% yttria, and the Vickers hardness and fracture toughness of the fabricated parts are close to the common ceramics prepared by conventional methods [21].

In this study we tried to fabricate fully dense ZrO_2_ via the DLP technique and evaluate the mechanical properties of DLP-fabricated ZrO_2_ using three-point bending tests with the digital image correlation (DIC) method, scanning electron microscopy (SEM) observations and fractography analysis. Defects are unavoidable in the ceramic body formed by the DLP method, related to the binder’s physical and chemical changes and corresponding reaction products during the sintering process [22]. Therefore, we discuss the crack nucleation and propagation mechanism in the debinding-sintering process before the conclusions.

## 2. Methodology

### 2.1. Materials and Slurry Preparation

One of the critical steps in the DLP method is to prepare a photo-curable slurry with characteristics that meet the printing requirements (such as not having a high viscosity and appropriate curing characteristics). The raw materials listed in Table 1 were used in this experiment and the powder composition as per the manufacturer is listed in Table 2.

HDDA is one of the most popular acrylate monomers, showing good agreement between low viscosity, mechanical resistance (due to cross-linking), and diffusion-controlled degradation during debinding. However, its high polymerization shrinkage creates internal stresses during curing, which leads to delamination and brittle behavior of the part [1]. Better results were achieved when HDDA was combined with monomers of higher functionality for enhancing cross-linking, for example TMPTA and TPGDA. TPO was used as the photoinitiator. DisperBYK (BYK-180) was the dispersant used to reduce powder agglomeration.

**Figure 1:**
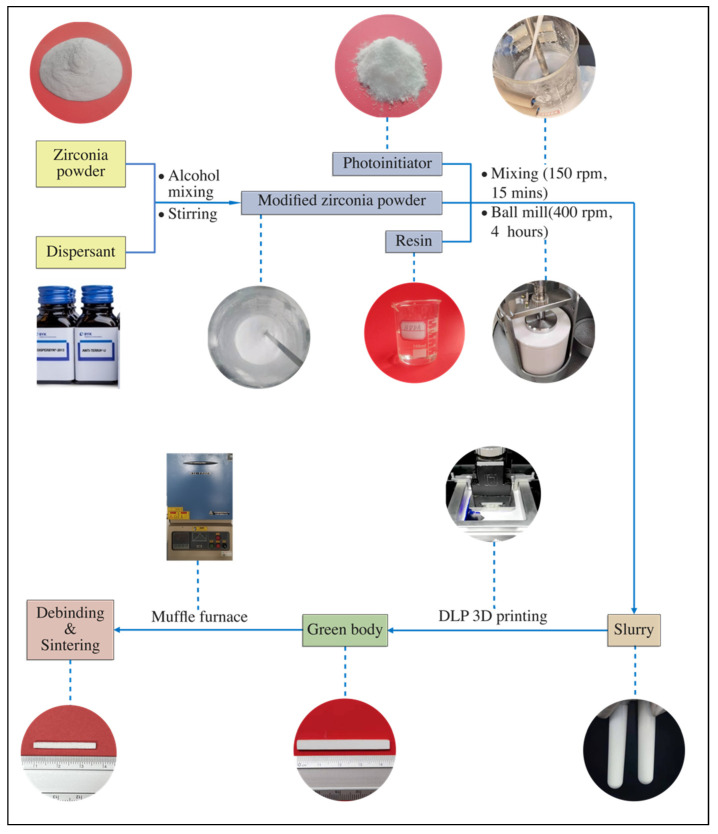
The fabrication process of DLP 3D printing ZrO_2_.

The process of powder modification and preparation of slurry is presented in Figure 1 and can be summarized as follows:

➢ Powder modification
  - The original ZrO_2_ powder was humid, so it should be placed in an oven (D2F6020AB, Tianjin Gongxing Laboratory Instrument Co., Ltd., Tianjin, China) to dry thoroughly.
  - Based on the mass of ZrO_2_ powder, 3 wt.% of DisperBYK (BYK-180, an alkylol ammonium salt of a copolymer with acidic groups) [23] was dissolved in ethanol solution by magnetic stirring at 60 °C.
  - Then the dried ZrO_2_ powder was added gradually into the DISPERBYK solution
  - The resulting ZrO_2_ slurry was magnetically stirred in a 60 °C water bath for 1 h to promote the adequate adsorption of the dispersant on the surface of the ZrO_2_ particles.
  - The modified ZrO_2_ powder was dried at 70 °C for 24 h and then screened through a sieve.
➢ Slurry preparation
  - First, 1,6-hexanediol diacrylate (HDDA, 70 g), tripropylene glycol diacrylate (TPGDA, 33 g) and 2,4,6-trimethylbenzoyldiphenyl phosphine oxide (TPO, 1g) were mixed to obtain a homogeneous photosensitive resin.
  - Next, an appropriate amount (55 wt.%) of the modified ZrO_2_ powder was added to the prepared photosensitive resin, and stirred with a mixer (GZ120-S, Shanghai Lilei Instrument Technology Co., Ltd., Shanghai, China) for 15 min.
  - Then the stable ZrO_2_ slurry was processed using a planetary ball mill for 4 h with a speed of 400 rpm (KE4, Ruiru Tec, Guangzhou, China) with a mass ratio of ZrO_2_ grinding balls to ZrO_2_ powder of 2:1 (grinding balls with three different sizes—7/5/3mm—were used to achieve better mixing).

### 2.2. DLP Procedures

**Figure 2:**
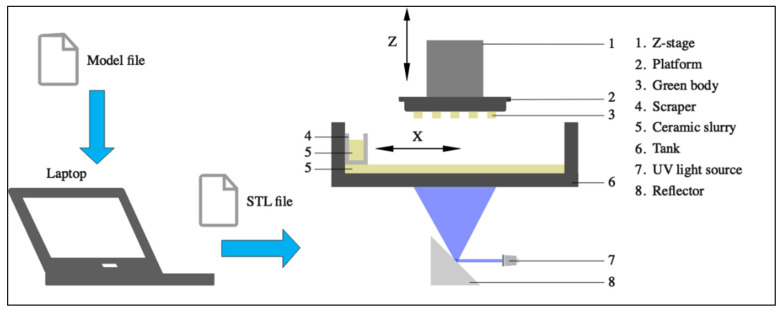
The schematic diagrams of DLP 3D printing procedures.

ZrO_2_ samples were printed using a DLP 3D printer (AUTOCERA-M -Beijing TenDimensions Technology Co., Ltd. Beijing, China). The .stl file was created by using the software provided by TenDimensions, which is compatible with the 3D printer type. First, the prepared ceramic slurry was poured into the scraper slot and spread slurry on the Teflon film. Then, UV light was exposed to the Teflon film surfaced, and the slurry was polymerized and cross-linked to form a single layer. After that, the Z-stage moved upwards, and the slurry was recoated on the Teflon film surface by scraper moving in the X-direction. Finally, this procedure was repeated to print the whole part. Figure 2 schematically shows the DLP process.

In DLP printing, curing thickness is a significant parameter determining the thickness of available layers and printing quality. Therefore, appropriate exposure times and light intensity must be found to ensure good bonding between cured layers during printing. After the preliminary test, the exposure time was determined as 2 s with a light intensity of 18 mW/cm^2^, and the average curing thickness was measured as 35 μm.

The printed samples were cleaned by ultrasonic cleaning in deionized water with resin cleaner in order to remove the remaining slurry on the printed samples, and then dried at 60 °C for 24 h.

### 2.3. Debinding and Sintering

After printing the green parts, a debinding process must be performed to remove excess resin from the green body. The debinding behavior depends on several factors, such as resin composition, component geometry, and solid loading. For these reasons, the thermogravimetric analysis (TGA) and differential thermogravimetry (DTG) of the cured slurry were performed and carried out on a TG/DSC3+ thermogravimetric analyzer (Mettler Toledo, Zurich, Switzerland).

Printed specimens must be sintered as fast as possible to avoid any potential negative effects of the environmental conditions on the mechanical properties of the ceramics. Therefore, a one-step sintering method was adopted after trial and experiment since the samples became exceptionally fragile after debinding. The debinding and sintering process was completed in a traditional muffle furnace (KSL-1700X, Hefei Kejing Material Technology Co., Ltd., Hefei, China). The sintering process is described in detail in the Section 3.

**Figure 3:**
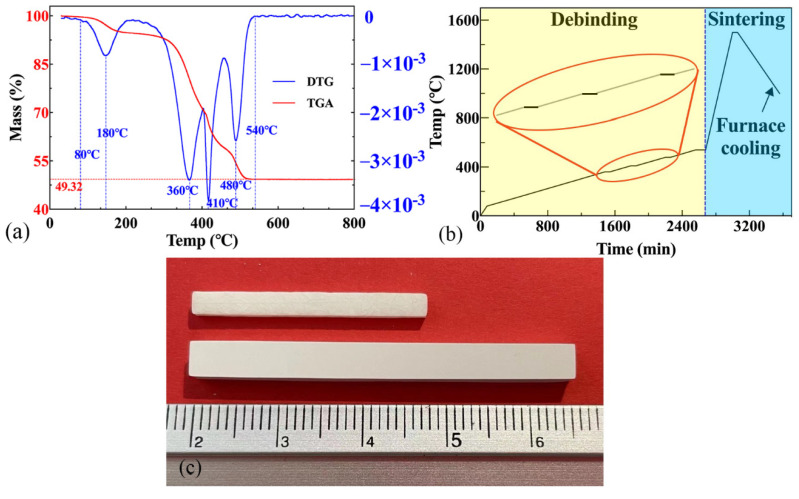
(**a**) The TG/DTG curves, (**b**) the debinding and sintering profiles of the green body and (**c**) the samples of before (below) and after (top) sintering.

Figure 3a illustrates the TG-DTG curves of the green body obtained after DLP printing. The cured resin began to decompose at 80 °C, and this finished at 540 °C. There were four prominent decomposition peaks around 180 °C, 360 °C, 410 °C, and 480 °C. The debinding process was designed according to the TG-DTG curve. The resin decomposed quickly between 180 °C and 410 °C during the debinding process. The four peaks represent the maximum thermal decomposition rates.

Figure 3b shows the temperature scheme used in the debinding and sintering process. In the debinding sintering process, the pores among the powders were squeezed and eliminated, and a highly dense component was formed.

In the low-temperature stage (debinding stage), the rapid expansion of gas would accelerate the initiation and growth of cracks [22]. Thus, the heating rate between 80 °C and 540 °C should be delicately controlled: various heating rates of 1 °C/min and 0.2 °C/min were implemented for the temperature range of RT–80 °C and 80–540 °C.

Meanwhile, during the debinding process in order to assure the fully chemical decomposition, the sample was retained one hour at temperatures of 180 °C, 360 °C, 410 °C, 480 °C, and 540 °C (prominent decomposition peaks), respectively. Moreover, a heating rate of 5 °C/min was selected for the entire sintering process up to 1500 °C, and kept for another 200 min. Figure 3c shows the printed samples before and after the sintering process.

### 2.4. Flexural Strength Tests

**Figure 4:**
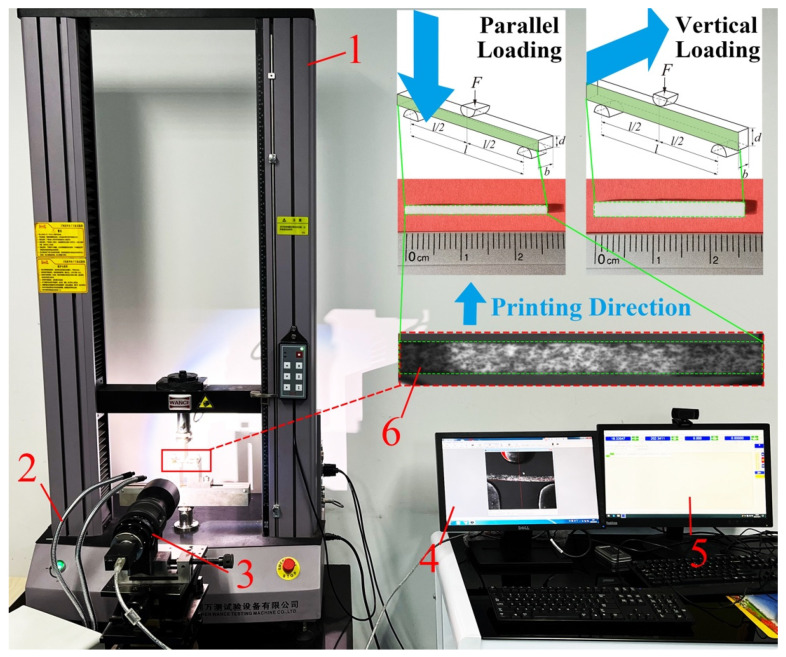
Three-point bending test facility (1—universal testing machine, 2—light source, 3—DIC camera, 4—DIC camera data acquisition, 5—testing machine data acquisition, 6—specimen).

The dimensions of the specimens were 27.26 (±0.09) mm × 2.43 (±0.04) mm × 1.70 (±0.01) mm (length, width, thickness). The specimens’ mechanical behavior is largely dependent on the applied fabrication process, strain rate, and material type. According to ASTM C 1161-02c (2008), the three-point bending test (Figure 4) was carried out on a universal testing machine (ETM104B, WANCE, Shenzhen, China). The three-point bending span was 20 mm, and the loading rate was 0.3 mm/min. Before testing, the samples were visually inspected to exclude artificial defects, the support structures were removed, and no further surface finishing was implemented after printing and cleaning. The mechanical test was terminated when the sample failed, and the maximum load obtained was used to calculate the flexural strength (*σ_f_*) and elastic modulus (*E_f_*) [24]:

(1) $$\sigma_{f}=\frac{3PL}{2bd^{2}}$$

(2) $$E_{f}=\frac{PL^{3}}{4bd^{3}v_{f}}$$

where *P*, *L*, *b*, *d* and *v_f_* are the maximum load (N), support span (mm), the width of the tested beam (mm), the thickness of the tested beam (mm) and the deflection at fracture (mm), respectively.

### 2.5. Characterization

DIC technology (The principle of DIC technology can be seen in Appendix A) was used to analyze the cracking process of the sintered ZrO_2_ samples under the three-point bending test. The surface of the samples was photographed at a speed of 3 frames per second by a CDD camera (GRAS-50S5M/C, FLIR Systems Inc, Wilsonville, OR, USA) with a pixel resolution of 5 million pixels. To obtain high-quality images measured by digital image correlation (DIC), two high-power LED lamps (95 W) were used as light sources and provided adequate illumination intensity.

The slurry used in the DLP printing process should have high fluidity to ensure the printing of ceramic parts with complex geometry within a reasonable processing time, meaning the slurry viscosity should not be too high. A rheometer (Physica MCR301, Anton Paar, Graz, Austria) was used to study the rheological behavior of the ceramic slurry. The functional relationship between viscosity and shear rate was measured in the range of 0–200 s^−1^ under isothermal conditions (25.0 °C).

The shrinkage rate of sintered samples was calculated using the expression $\delta =(l_{0}-l_{1})/l_{0}\times 100\%$ (where *l*_0_ and *l_1_* stand for the length before and after sintering, respectively), and the shrinkage rate *δ* was measured via a Vernier caliper. The apparent density and open porosity of the sintered sample were determined by True Density Analyzer (AccuPyc II 1345, Micromeritics Instrument Ltd., Atlanta, GA, USA). Vickers hardness was determined using the Vickers indentation method at an indentation load of 9.8 N for 15 s (HMV-2TADW, Shimadzu Corporation, Kyoto, Japan). For phase identification, an X-ray diffractometer (XRD) (AXSD2 Advance, Bruker, Billerica, MA, USA) was utilized. The microstructures and the fracture surfaces of samples were examined using the SEM (SU100, Hitachi, Tokyo, Japan).

## 3. Results and Discussion

### 3.1. Rheological Properties

**Figure 5:**
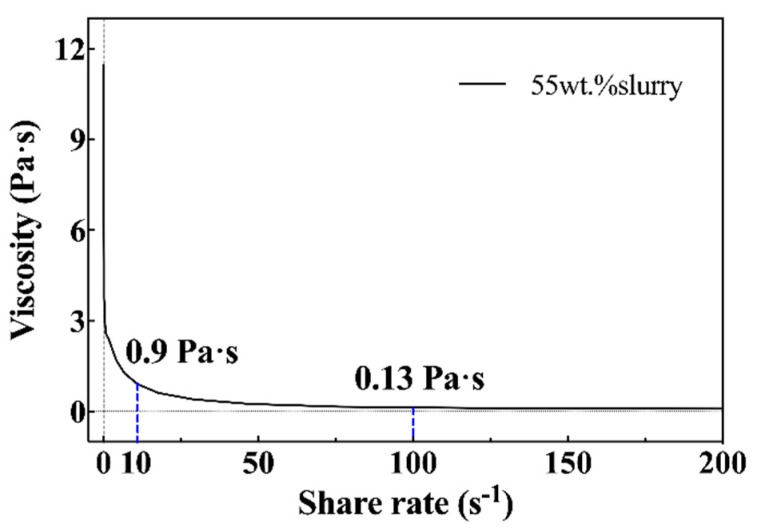
Shear viscosity (η) of ZrO_2_ slurry with respect to the shear rate in the range of [0, 200] (s^−1^).

The viscosity of 3 Pa·s at a shear rate of 10s^−1^ is generally considered to be the upper limit of processable slurry [25]. Figure 5 shows the rheological properties of the ZrO_2_ slurry. The viscosity at 10 s^−1^ and 100 s^−1^ was 0.9 Pa·s and 0.13 Pa·s, respectively. The prepared slurry revealed shear-thinning fluid behavior, indicating the slurry was appropriate for printing processing.

### 3.2. Physical Properties of the Sintered Samples

The insets in Figure 4 show the sintered sample’s front surface (along the printing direction) and the top surface (perpendicular to the printing direction). Good flatness can be observed on the top surface, and this uniform pattern shows the precision of the printing process.

The average apparent density *ρ_a_* of sintered ZrO_2_ samples was 5.65 g/cm^3^, while the true density *ρ_t_* of sintered ZrO_2_ samples was 5.98 g/cm^3^, thus the relative density *ρ_r_* of sintered ZrO_2_ samples ($\rho_{r}=\rho_{a}/\rho_{t}\times 100\%$) is 94.48%. This relative density was lower than that of other samples (99%) prepared by the DLP method [21], which resulted in a lower Vickers hardness value (1128 ± 40 HV) compared to the sample with higher relative density (~1190 HV) [21].

The variation of the anisotropic linear shrinkage of the sintered specimen is listed in Table 3. Due to the friction between the specimen and crucible during sintering, the linear shrinkage in the width (*b*) direction was slightly lower than that in the thickness (*d*) direction.

### 3.3. XRD 

**Figure 6:**
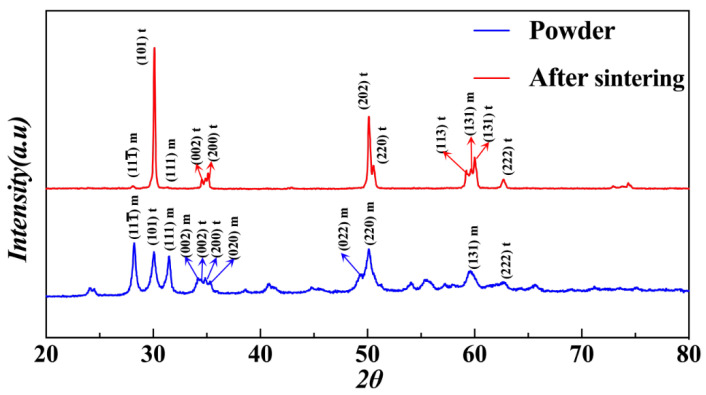
The XRD pattern of the original ZrO_2_ powder and the sintered ZrO_2_ sample.

Crystalline phases of m-ZrO_2_ and t-ZrO_2_ were both detected in the original ZrO_2_ powder because 3 mol% Y_2_O_3_-stabilized ZrO_2_ powder was used in the present study (Figure 6). However, after sintering up to 1500 °C, the amount of m-ZrO_2_ was below the detection limit in the specimen due to the m→t phase transition. As a result, the prominent peaks of 2θ at 30.2°, 34.7°, and 35.2° became more significant (Figure 6). These three peaks corresponded to the characteristic crystal planes (101)t, (002)t, and (110)t of t-ZrO_2_, respectively.

### 3.4. Flexural Properties

**Figure 7:**
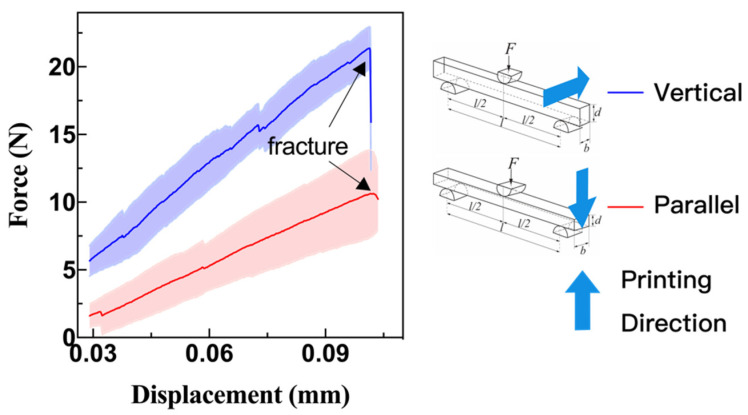
The flexural strength curves.

The force-displacement curves of the samples under parallel and vertical bending loads (with respect to the printing direction) are shown in Figure 7. The flexural strength and the corresponding elastic modulus under parallel loading were 56.63 ± 3.97 MPa and 199.15 ± 12.51 GPa, respectively. While under vertical loading, both the flexural strength and the related elastic modulus were higher than that under parallel loading, which were 70.98 ± 6.62 MPa and 205.07 ± 16.88 GPa, respectively.

Although the elastic modulus values are close to those of partially stabilized ZrO_2_ fabricated by the conventional sintering method [26], there is still a certain gap between the flexural strength in this study and better levels reported in other literature [16]. One of the key reasons is the existence of surface defects. It should be noted that no surface treatment was used in the present study in order to maintain the as-fabricated properties, so surface defects were unavoidable and resulted in crack initiation points. If the surface defects are present at any point during the three-point bending test, the specimen tends to break quicker than the solid portion, and the flexural strength decreases. Moreover, the internal cracks and delamination formed during debinding and sintering are also the dominant phenomena affecting the flexural strength. This will be discussed in detail later in Section 3.5 together with the SEM images.

**Figure 8:**
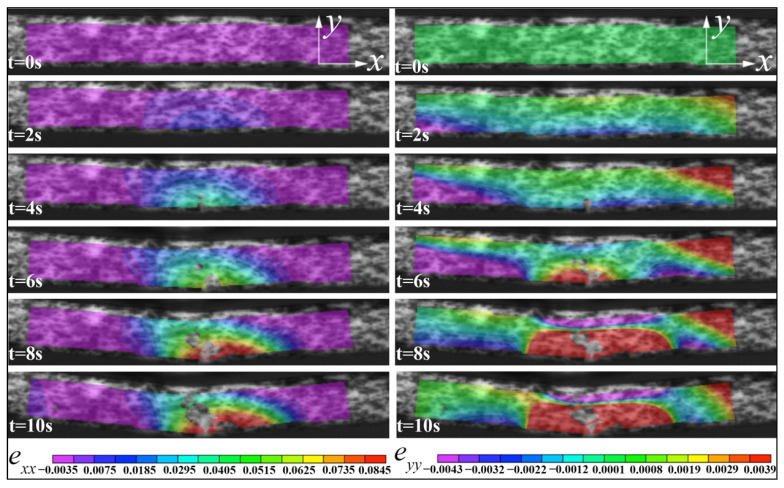
The DIC images of the horizontal strain (*e_xx_*) and the vertical strain (*e_yy_*) of ZrO_2_ samples under a three-point parallel bending test.

**Figure 9:**
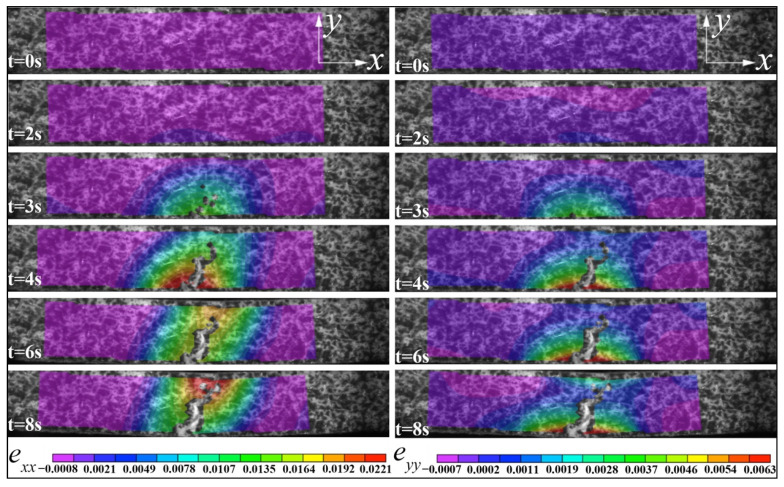
The DIC images of the horizontal strain (*e_xx_*) and the vertical strain (*e_yy_*) of ZrO_2_ samples under a three-point vertical bending test.

The measured horizontal (*e_xx_*) and vertical strain (*e_yy_*) on the surface of the specimen are given in Figure 8 for the parallel loading and Figure 9 for the vertical loading. Regarding horizontal strain (*e_xx_*), the maximum strain (*e_pxx_max_*) area always appeared at the bottom center before the failure for the parallel loading case (Figure 8), but the maximum strain (*e_vxx_max_*) area for the vertical loading case moved upward from the bottom center to the top center (Figure 9). For the vertical strain contours (*e_pyy_*) of the sample under parallel loading, there was a clear dividing line between the minimum strain (*e_pyy_min_*, compressive) and the maximum strain (*e_pyy_max_*, tensile), and the tensile zone was more extensive than that of the compressive zone. However, this distinction line did not appear in the vertical loading case (Figure 9). The sample’s vertical strain (*e_vyy_*) under vertical loading declined from the bottom to the top along the crack path.

It can be seen that in the case of parallel loading, mainly the bottom printing layer was stretched to both lateral sides (Figure 8). Because the tensile strength of ceramic is typically much lower than the compressive strength [27], the failure started at the bottom first. Under vertical loading, the bottom dense layers were thicker than that of samples under parallel loading; that’s why the vertical bending strength was higher than that obtained in the parallel bending test.

Under parallel loading, the interlayer strength plays a dominant role in the flexure strength and fracture mode. On the contrary, the in-plane strength of printing layers determines the deformation behavior of samples under vertical loading. Because the interlayer gaps intervened in the propagation of cracks, the crack propagated through the thickness direction (under parallel loading) was slower than that propagated through the width direction (under vertical loading). A detailed fractography analysis will be given in the next section combined with the SEM observations.

### 3.5. SEM

**Figure 10:**
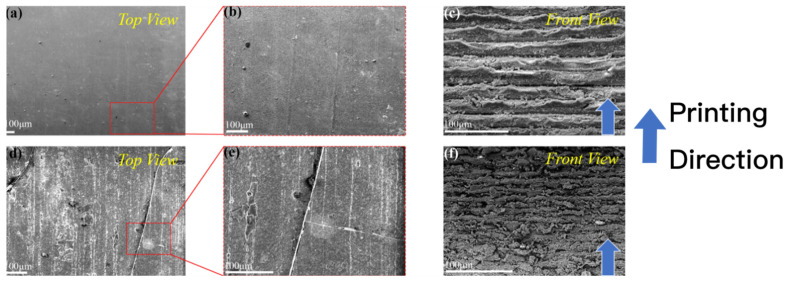
The SEM images of 3D printing ZrO_2_ specimen. (**a**–**c**): before sintering; (**d**–**f**): after sintering.

**Figure 11:**
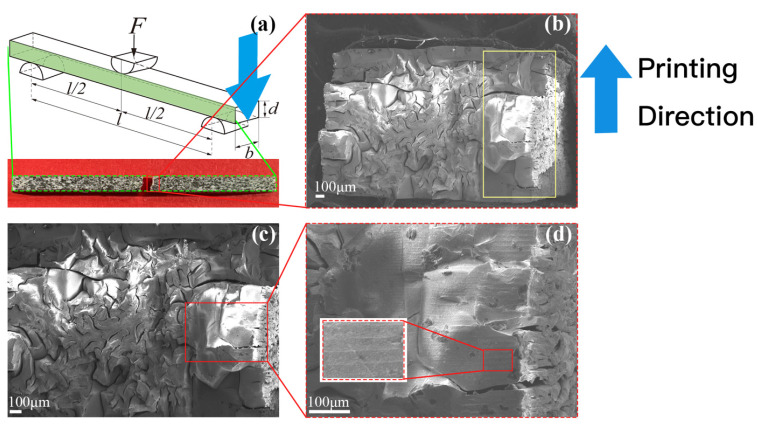
The SEM images of three-point parallel loading. (**a**): Schematic diagram of the experiment; (**b**–**d**): Fracture morphology and its partial enlarged views.

**Figure 12:**
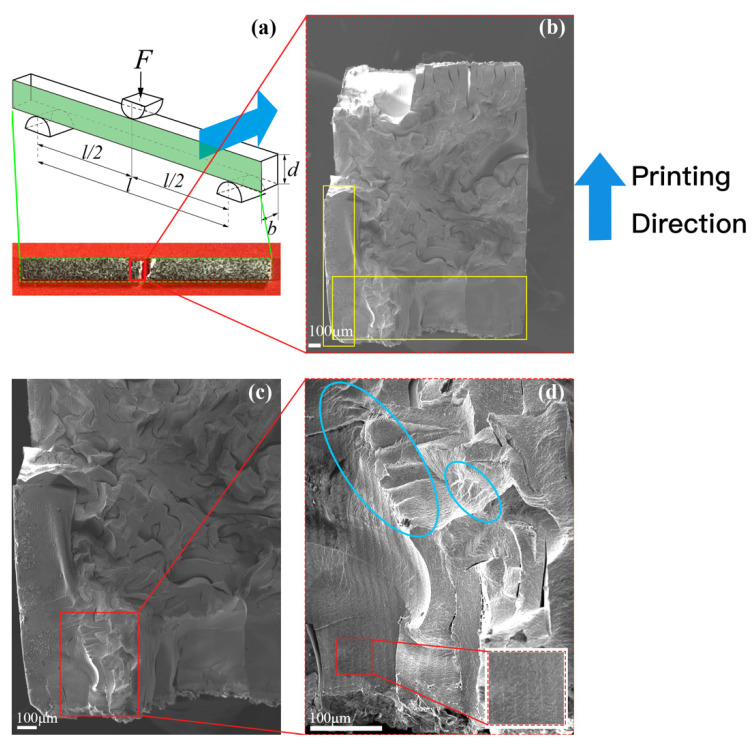
The SEM images of three-point vertical loading. (**a**): Schematic diagram of the experiment; (**b**–**d**): Fracture morphology and partially enlarged views.

The SEM analysis can provide additional evidence for the microstructure of the ZrO_2_ samples (Figure 10) and the fracture morphology (Figure 11 and Figure 12). Before sintering (green body), the top surface was flat and smooth without any visible cracks (Figure 10a,b); however, after sintering (brown body), the top surface became coarse due to the shrinkage effect, and cracks were observed (Figure 10d,e). Moreover, the front surface exhibited a clear lamination along the printing direction (Figure 10c,f), in which the interlayer was thinner after sintering due to shrinkage. A suitable surface treatment would improve the surface quality as well as the flexural strength of 3D printing ZrO_2_ specimen. For ZrO_2_, sandblasting and wet-grinding have been recommended to create a surface region of compressive stresses, which increases the mean flexural strength of ZrO_2_ [23,28].

The fracture morphology and its partially enlarged views for paralleling and vertical bending tests are displayed in Figure 11 and Figure 12, respectively. Although the samples appeared to be stacked layer by layer from the outside, they were actually interlaced with irregular and winding cracks, indicating the debinding and sintering process has a prominent influence on the interior microstructure. We must admit that the flexural strength was around 20 times lower than that of common well prepared 3Y-TZP samples (i.e., 1–1.2 GPa of flexural strength) because so cracked sintered samples were obtained.

**Figure 13:**
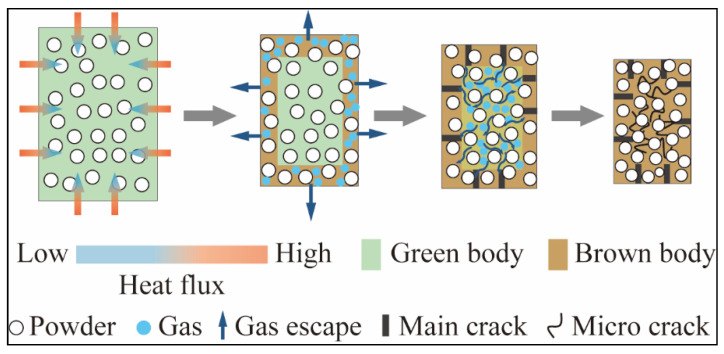
The schematic diagrams of crack initiation during the debinding and sintering process.

The sintered ZrO_2_ specimen was quite dense at depths of only a few hundred microns (as pointed out with yellow rectangles in Figure 11b and Figure 12b), and this dense layer along the width direction was thicker than in the printing direction. Moreover, the interior of the ZrO_2_ specimen was filled with needle-like cracks in 2D view, but probably more like a frisbee in 3D view. The intense cracks indicate that the thermal diffusion in the debinding and sintering process and the mismatch of thermal expansion coefficient between resin (organic matter) and solid phase material determine the final microstructure. Figure 13 schematically shows the crack initiation mechanism during the debinding and sintering process. The green body was heated following the temperature profile shown in Figure 3b. At the decomposition stage the organic matter probably was fluid and only some interconnected micro-channels were formed, and there is only a tiny amount of densification. The gasified binder escaped from the out layers, resulting in the dense outer layers and interlayer straight cracks (main cracks) at the beginning of the sintering stage. The volume of the green body decreased with the increase of the temperature, and the gas was discharged from inside to outside through the interconnected curved channels (micro cracks), corresponding to the shrinkage of the printed samples.

The intense cracks are mainly due to the low solid loading of ZrO_2_. The slurry contains 55 wt% of ZrO_2_, which equals a volume percentage of 17 vol.% (see Appendix B for details). A ceramic loading greater than 40 vol% is necessary for avoiding defects during post-processing, such as delamination and cracks. The dispersant in the slurry is a key factor that affects the maximum solid loading of ZrO_2_.

ZrO_2_ powder has a strong tendency to agglomerate due to the Van der Waals forces, thus increasing the suspension viscosity. Dispersants can reduce the Van der Waals potential and improve the manufacturability of highly loaded ceramic slurries. The dispersant we used, named DisperBYK-180, is a commercial hyperdispersant based on non-polar acrylates. When used with acrylates, oleic acid shows better dispersion than stearic acid and sebacic acid [29]. Its unsaturated chain seems to have a better affinity with the double bonds of non-polar acrylates.

Moreover, the amount of dispersants is crucial to obtain an optimum viscosity. A low amount of dispersant can result in the flocculation due to an incomplete coverage of the particle surface. On the contrary, a large amount of dispersant above the adsorption limit increases the suspension viscosity [30].

The complex microstructure, including surface defects, micro-cracks and delamination, significantly influence the flexural strength of the printed ZrO_2_ specimen as displayed in Figure 7. A better understanding of the additive formulation (particularly the dispersant), the curing process itself and the debinding-sintering process are necessary for future development [31]. In that case, the DLP method can be comparable to other advanced ceramic processing techniques by fabricating structural products (i.e., bone scaffold).

The crack shape was different inside and outside. The surrounding dense part was mainly composed of large and straight cracks between printing layers, whereas the interior contained numerous small winding cracks (Figure 11b, Figure 12b and Figure 13). The relatively smooth fracture surface in the outside layers (yellow rectangles in Figure 11 and Figure 12) is highly possible due to an intergranular fracture in a polycrystalline material with submicron grains size. In the next step, the SEM analysis with a higher resolution is the highest priority that needs to be undertaken to clarify the fracture mode.

**Figure 14:**
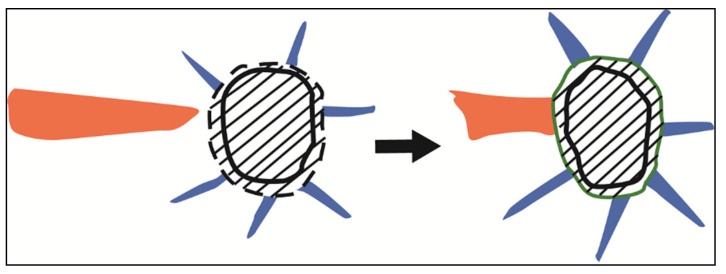
The schematic diagrams of crack propagation.

The fracture morphology was characterized by a transition from a relatively smooth area in the outer layer to a few stepped areas (blue ellipses) and then an abundant number of bending cracks in the interior. When the big cracks propagate and meet the smaller cracks, the crack tip would be weakened, and the big crack may change its propagation direction and decompose into several small cracks (Figure 14). As a result, the crack propagation would slow down, and some cracks may even be compressed and closed. Due to the crack deflection toughening mechanism, the fracture toughness of DLP-fabricated ZrO_2_ was improved.

In the present experiments, the size of the fully dense part ranges between 200 to 300 μm, but we must admit that our experimental samples have many imperfections, resulting in lower mechanical properties. Other related research has shown that the size of the fully dense part can be higher. Osman et al. used DLP to fabricate a custom-designed implant with an excellent dimensional accuracy [3]. The dental implant is about *ϕ*4b × 18 mm, but the micro-cracks, porosities and interconnected pores were still unavoidable. Schönherr et al. [4] printed the glass ceramics dental crowns with a high printing precision and a high reproducibility, however, they did not conduct a crack analysis. In general, DLP is more suitable for printing relatively small pieces, for example dental bridges, crowns, inserts and implants. In addition, the DLP technique might be more suitable for preparing spatial lattice structures with limited thickness to give full play of their strength rather than continuum bulk materials. For example, researchers have designed several ceramic bone scaffolds for bone regeneration [32,33,34,35].

## 4. Conclusions

ZrO_2_ parts have been fabricated using a DLP 3D printing technology in which an attempt has been made to investigate its three-point bending deformation mechanism via DIC, SEM and fractography. The main conclusions are summarized as follows:

- Both the flexural strength and the related elastic modulus of the samples under vertical loading were higher than that under parallel loading. That is because under parallel loading the interlayer strength plays a dominant role; whereas the in-plane strength of printing layers determines the deformation behavior of samples under vertical loading.
- The horizontal *e_pxx_max_* always appeared at the bottom center before the failure for the parallel loading case, but the *e_vxx_max_* for the vertical loading moved upward from the bottom center to the top center. There was a clear dividing line between the perpendicular *e_pyy_min_* and the *e_pyy_max_* under parallel loading; however, the *e_vyy_* declined from the bottom to the top along the crack path under vertical loading.
- The intense cracks are mainly due to the low solid loading of ZrO_2_, indicating the additives formulation used was not adequate. The fracture morphology was characterized by a transition from a relatively smooth area in the outer layer to a few stepped areas and then the interior with numerous micro-cracks.
- The relatively smooth fracture surface in the outside layers is highly possible due to an intergranular fracture in a polycrystalline material with submicron grains size. A higher resolution SEM analysis is necessary to clarify the fracture mode.
- The differences in internal and external microstructures reflected that DLP technology might be more suitable for preparing spatial lattice structures with limited thickness to give full play of zirconia strength (i.e., ceramic bone scaffolds for bone regeneration). In the next work, the influence of the sample dimension on the mechanical property should be investigated.

## Figures and Tables

**Table 1:** Slurry constituents and their properties.

Constituents		D_50_ /nm	Molar Mass /g mol^−1^	Viscosity /mPa·s	Density /g cm^−3^	Supplier
Powder	Yttria-stabilized ZrO_2_	500	—	—	6.05	Jiangsu FREDS Powder Technology Co. Ltd., Yixing, China
Resin	HDDA	—	226	5–10	1.01–1.03	Yinchang Xin Cailiao Ltd., Shanghai, China
	TPGDA	—	300	10–15	1.03	
Photoinitiator	TPO	—	—	—	1.2	
Dispersants	BYK-180	—	—	—	1.16	BYKGardner GmbH, Geretsried, Germany

**Table 2:** Material composition as per manufacturer.

Powder Composition	
Y_2_O_3_ (mol%)	3
D_50_ (nm)	500
ZrO_2_ (wt.%)	94.7
Y_2_O_3_ (wt.%)	5.2 ± 0.2
Al_2_O_3_ (wt.%)	≤0.01
SiO_2_ (wt.%)	≤0.01
Fe_2_O_3_ (wt.%)	≤0.01
CaO (wt.%)	≤0.005
MgO (wt.%)	≤0.005
TiO_2_ (wt.%)	≤0.002
Na_2_O (wt.%)	≤0.005
Cl^−1^ (wt.%)	≤0.01
Specific Surface Area (m^2^/g)	6–8
Size of the crystallite (nm)	150

**Table 3:** Shrinkages of the samples.

Direction (*n* = 13)	Shrinkage (%)	Mean + SD *	Mean + SEM ^†^
Thickness, *d*	41.90	0.77	0.21
Width, *b*	41.58	0.82	0.23
Average	41.74	0.50	0.14

* SD: Standard Deviation **^†^** SEM: Standard Error of Mean.

**Figure A1:**
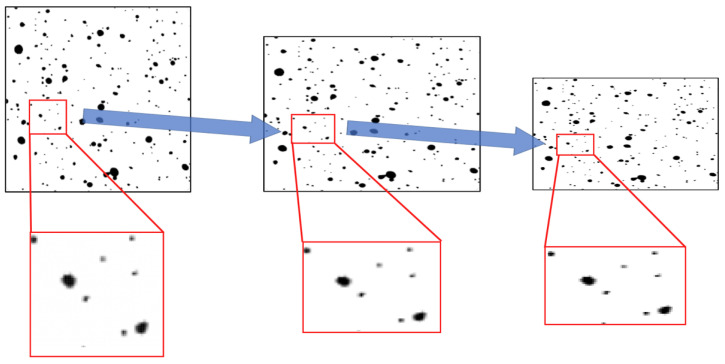
Schematic illustration of DIC subset tracking before and after deformation.

**Figure A2:**
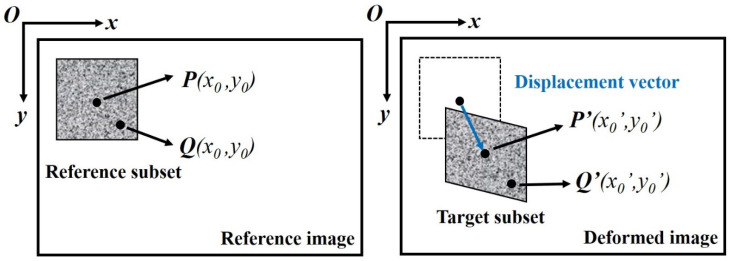
Schematic illustration of a reference square subset before deformation and a target (or deformed) subset after deformation.

**Figure A3:**
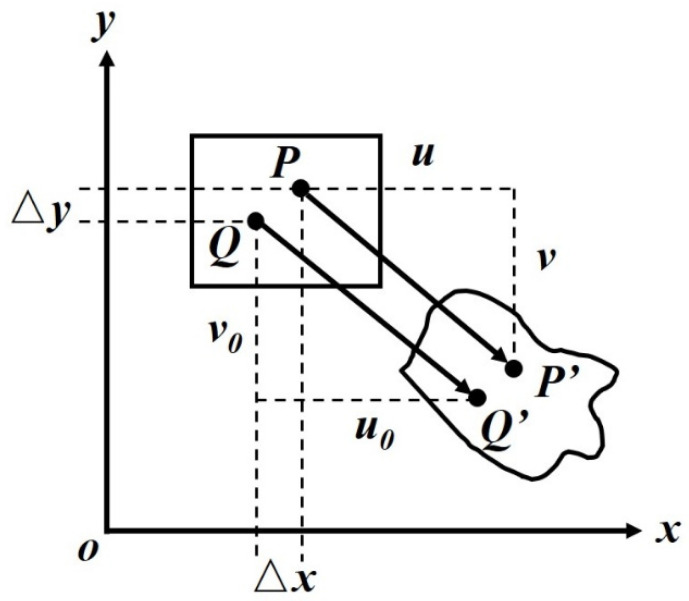
Schematic illustration of displacement analysis.

**Figure A4:**
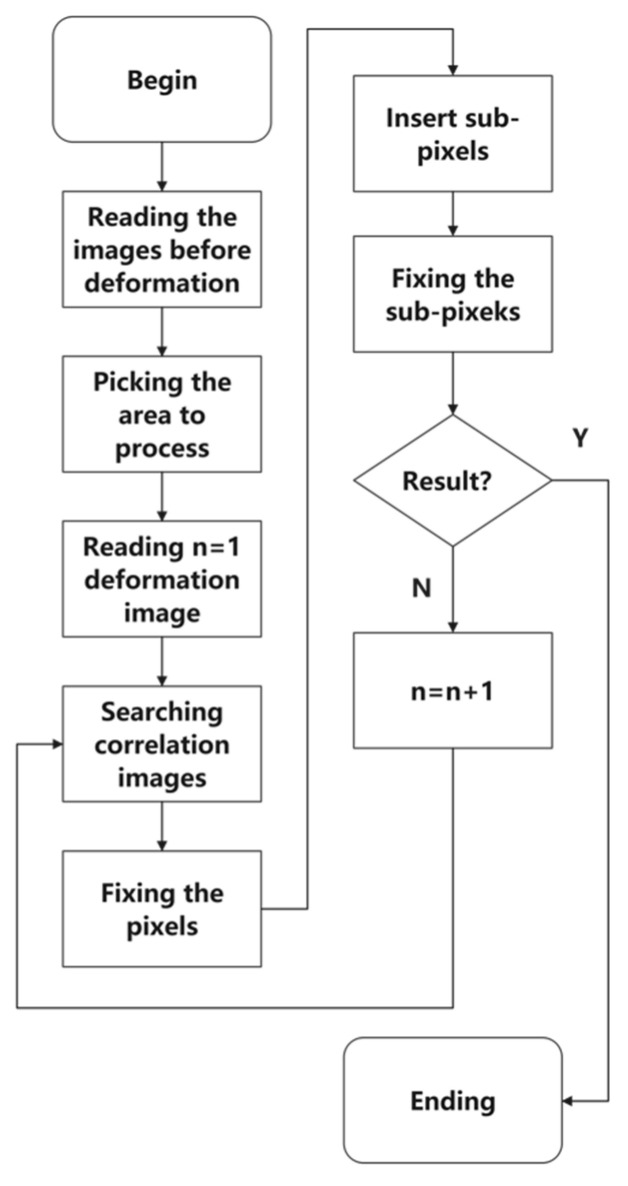
DIC correlation analysis flowchart.

## Data Availability

This study did not report any data.

## Appendix A

The basic principle of the digital image correlation (DIC) measurement method is based on an image with a certain distribution of feature points (called speckle map), these feature points are pixel points used as coordinates, and the grayscale of pixels is used as an information carrier, before the correlation algorithm runs, to select a square image sub-area, and the center of this sub-area is the pixel of interest. In the process of image movement or deformation, the displacement vector at the center point of the sub-area can be obtained by tracking the position of the image sub-area in the deformed image (that is, the target image, as shown in Figure A1). After analyzing the displacement vectors of the center points of multiple sub-regions, the displacement field of the entire analysis region is formed.

As shown in Figure A2, in the reference image, the center point *P*(*x_0_, y_0_*) of the sub-area and any adjacent point *Q*(*x_i_, y_i_*) in the sub-area are *P*’(*x’, y’*), *Q*’(*x’, y’*) in the target subregion of the deformed image. A series of neighbors in the reference subregion are still neighbors of the center point in the target subregion, due to the continuity assumption of deformation.

Figure A3 shows the schematic diagram of displacement analysis. For point *P*’, the following formula holds:

$$x_{0}^{\prime }=x_{0}+u\;y_{0}^{\prime }=y_{0}+v$$

where *u*, *v* are components of the point *P*’ displacement at axis *x*, *y*.

For point *Q*′, the following formula holds:

$$x_{i}^{\prime }=x_{i}+u_{Q}\;y_{i}^{\prime }=y_{i}+v_{Q}$$

where *u_Q_*, *v_Q_* are components of the point *Q*’ displacement at axis *x*, *y*.

Before the deformation point *Q*’s gray value can write as:

$$f\left(Q\right)=f\left(x_{i},y_{i}\right)\;g\left(Q^{\prime }\right)=g\left(x_{i}^{\prime },y_{i}^{\prime }\right)$$

where *f* and *g* respectively represent the gray distribution of the two frames of images recorded before and after deformation.

If:

$$u_{Q}=u+u_{x}\Delta x+u_{y}\Delta y\;v_{Q}=v+v_{x}\Delta x+v_{y}\Delta y\;\Delta x=x_{i}-x_{0},\Delta y=y_{j}-y_{0}$$

*P* = [*u u_x_ u_y_ v v_x_ v_y_*]^T^ were the unknown vectors in the formula, including the displacement variable.

In order to accurately determine the displacement vector, it is necessary to determine the unique correspondence between the target sub-area and the reference sub-area, and introduce the concept of correlation coefficient (or correlation criterion) C(*f*, *g*) as a measure of the similarity between two images. Mathematical indicators.

$$C\left(f,g\right)=C\left(x_{i},y_{i},x_{i}^{\prime },y_{i}^{\prime }\right)=C\left(P\right)$$

That is, the correlation coefficient is a function of P. By finding the minimum value of the correlation coefficient:

$$\frac{\partial C}{\partial P_{i}}=0$$

the *P* value can be determined through a certain iterative algorithm. Its iterative algorithm includes two processes of integer pixel search and sub-pixel search. The analysis process is shown in Figure A4. By searching the sub-regions of the whole field, the same sub-region before and after deformation can be uniquely determined. At this time, the displacement of the sub-region can be determined. The vector has also been determined, and the strain field can be determined from the relationship between displacement and strain.

## Appendix B

The mass content is used in this paper, and the conversion of mass content and volume content of zirconia can be calculated by the following formulas:

(A1) $$m_{TPO}=1\;g\;m_{HDDA}=70\;g\;m_{TPGDA}=33\;g\;m_{ZrO_{2}}=127.1\;g$$

(A2) $$wt.=\frac{m_{ZrO_{2}}}{m_{whole}}\times 100\%\;=\frac{m_{ZrO_{2}}}{m_{ZrO_{2}}+m_{HDDA}+m_{TPGDA+}m_{TPO}}\;\times 100\%\;=\;\frac{127.1}{127+70+33+1}\times 100\%\;=55\%$$

where 55wt.% ZrO_2_ slurry was composed of 127.1 g ZrO_2_ powder, 1 g *TPO*, 70 g *HDDA* and 33 g *TPGDA*. The density of ZrO_2_, *TPO*, *HDDA* and *TPGDA* was 6.05 g/cm^3^, 1.2 g/cm^3^, 1.01 g/cm^3^ and 1.03 g/cm^3^.

(A3) $$V_{ZrO_{2}}=\frac{m_{ZrO_{2}}}{\rho_{ZrO_{2}}}=\frac{127.1}{6.05}{\mathrm{cm}}^{3}\;=\;21.01{\mathrm{cm}}^{3}\;V_{HDDA}=\frac{m_{HDDA}}{\rho_{HDDA}}=\frac{70}{1.01}{\;\mathrm{cm}}^{3}=69.31{\;\mathrm{cm}}^{3}\;V_{TPGDA}=\frac{m_{TPGDA}}{\rho_{TPGDA}}=\frac{33}{1.03}{\;\mathrm{cm}}^{3}=32.04{\;\mathrm{cm}}^{3}\;V_{TPO}=\frac{m_{TPO}}{\rho_{TPO}}=\frac{1}{1.2}{\;\mathrm{cm}}^{3}=0.97{\mathrm{cm}}^{3}$$

(A4) $$vol.\%=\frac{V_{ZrO_{2}}}{V_{whole}}\times 100\%$$

(A5) $$vol.\%=\frac{V_{ZrO_{2}}}{V_{whole}}\times 100\%\;=\frac{V_{ZrO_{2}}}{V_{ZrO_{2}}+V_{HDDA}+V_{TPGDA}+V_{TPO}}\times 100\%\;=\frac{21.01}{21.01+69.31+32.04+0.97}\times 100\%\;=17.04\%$$
